# SNPs detection in *DHPS*-*WDR83* overlapping genes mapping on porcine chromosome 2 in a QTL region for meat pH

**DOI:** 10.1186/1471-2156-14-99

**Published:** 2013-10-08

**Authors:** Paolo Zambonelli, Roberta Davoli, Mila Bigi, Silvia Braglia, Luigi Francesco De Paolis, Luca Buttazzoni, Maurizio Gallo, Vincenzo Russo

**Affiliations:** 1 Dipartimento di Scienze e Tecnologie Agro-alimentari (DISTAL), Università di Bologna, Via Fratelli Rosselli, 107, 42123 Reggio Emilia, Italia; 2 Consiglio per la Ricerca e la sperimentazione in Agricoltura, Via Salaria 31, Monterotondo Scalo, 00015 Roma, Italia; 3 Associazione Nazionale Allevatori Suini (ANAS), via Lazzaro Spallanzani 4/6, 00161 Roma, Italia

**Keywords:** Swine, Meat pH, Single nucleotide polymorphism, *DHPS*, *WDR83*, Overlapping genes

## Abstract

**Background:**

The pH is an important parameter influencing technological quality of pig meat, a trait affected by environmental and genetic factors. Several quantitative trait loci associated to meat pH are described on PigQTL database but only two genes influencing this parameter have been so far detected: Ryanodine receptor 1 and Protein kinase, AMP-activated, gamma 3 non-catalytic subunit. To search for genes influencing meat pH we analyzed genomic regions with quantitative effect on this trait in order to detect SNPs to use for an association study.

**Results:**

The expressed sequences mapping on porcine chromosomes 1, 2, 3 in regions associated to pork pH were searched *in silico* to find SNPs. 356 out of 617 detected SNPs were used to genotype Italian Large White pigs and to perform an association analysis with meat pH values recorded in *semimembranosus* muscle at about 1 hour (pH1) and 24 hours (pHu) post mortem.

The results of the analysis showed that 5 markers mapping on chromosomes 1 or 3 were associated with pH1 and 10 markers mapping on chromosomes 1 or 2 were associated with pHu. After False Discovery Rate correction only one SNP mapping on chromosome 2 was confirmed to be associated to pHu. This polymorphism was located in the 3’UTR of two partly overlapping genes, Deoxyhypusine synthase (*DHPS*) and WD repeat domain 83 (*WDR83*). The overlapping of the 3’UTRs allows the co-regulation of mRNAs stability by a cis-natural antisense transcript method of regulation. *DHPS* catalyzes the first step in hypusine formation, a unique amino acid formed by the posttranslational modification of the protein eukaryotic translation initiation factor 5A in a specific lysine residue. *WDR83* has an important role in the modulation of a cascade of genes involved in cellular hypoxia defense by intensifying the glycolytic pathway and, theoretically, the meat pH value.

**Conclusions:**

The involvement of the SNP detected in the *DHPS/WDR83* genes on meat pH phenotypic variability and their functional role are suggestive of molecular and biological processes related to glycolysis increase during post-mortem phase. This finding, after validation, can be applied to identify new biomarkers to be used to improve pig meat quality.

## Background

Meat pH is an important parameter for the quality assessment of fresh and seasoned meat products [1]. The pH is also influenced by slaughter procedure as well as post slaughter carcass management and it is also under genetic control. Phenotypic variation of meat pH is partially regulated by genes as indicated in the review of [2] who reported 0.16 as the average heritability value for pH scored at about 1 hour post mortem (pH1) and 0.21 as the average value for pH recorded at 24 hours post mortem (pHu). Other researches showed that the heritability of pHu in Large White ranged from 0.29 to 0.45 [3,4]. Up to now only two major genes related to pig meat pH have been identified: Ryanodine receptor 1 (*RYR1*) mapped on *Sus scrofa* chromosome (SSC) 6 [5] and Protein kinase, AMP-activated, gamma 3 non-catalytic subunit (*PRKAG3*), located on SSC13 [6]. In addition to these evidences showing an effect of single genes, other research reported significant Quantitative Trail Loci (QTL) for meat pH in several porcine chromosomes as indicated in Pig QTL database (PigQTLdb) [7-9].

With the aim of searching genes responsible for QTL effect on pig meat pH, single nucleotide polymorphisms (SNPs) detected in the transcribed sequences of coding genes located on three QTL regions (QTLRs) of SSC1 (60–80 cM), SSC2 (55–66 cM) and SSC3 (42–60 cM), were utilized to perform an association analysis with meat pH values.

## Results and discussion

### SNPs detected in transcribed sequences located within the selected QTL regions

By multiple alignment of the sequences of the selected UniGene [10] entries we localised 2409 clusters containing both mRNAs and ESTs sequences and, after filtering as described in Methods, we retained 1822 clusters (Table 1). Among them we detected 353 UniGene clusters containing SNPs. On the whole 617 SNPs were found and, after removing those separated by less than 80 nucleotides, the remaining ones were 379 that decreased to 356 after checking their suitability to be used on the GoldenGate system with a score >0.6 (designability rank = 1).

**Table 1:** Summary of the steps utilized to identify the genotyped SNPs

**Porcine chromosome**	**No of porcine UniGene clusters whitin QTLR**	**No of selected porcine UniGene clusters**	**No of porcine UniGene clusters containing SNPs**	**No of useful SNPs within QTLR**
1	232	154	23	34
	224	157	41	53
	331	250	37	54
2	232	188	35	67
	563	444	83	104
3	425	340	61	35
	105	81	2	2
	297	208	71	30
Total	2409	1822	353	379

The position of the selected SNPs was precisely defined on porcine genome (version 10.2) allowing to align the location of the studied QTLR, based on the linkage map, to the physical map (Table 2). On SSC1 the examined region was 106.9-215.8 Mb, on SSC2 the considered segment was 32.7-77.9 Mb, and on SSC3 we analyzed the region 18.7-62.6 Mb. The SNP markers were placed within the chromosome regions detected by the search in PigQTLdb. For the SNP placement we used the currently most updated pig genomic sequence version 10.2 [11]. By comparing the physical length of the three chromosome portions and the number of SNPs detected within each of them we observed that SNPs are not evenly distributed because we searched all SNPs present in genes and no selection based or equal distribution was carried out in order to maintain as much polymorphisms as possible. The average interval between adjacent polymorphisms is less than 1 Mb. There are differences among chromosomes with intervals between adjacent SNPs ranging from 0.26 Mb on SSC2 to 0.77 Mb on SSC1. On the whole, the utilized approach for the detection of SNPs on transcribed sequences allowed to place approximately two to four markers for each cM, assuming that 1 cM corresponds, on average, to a segment of 1.3 Mb. This correspondence was obtained by comparing the total nucleotide length of the porcine genome of 3,024,658,544 nt reported on ENSEMBL web site [12] with the length of the linkage map reported by [13] represented by 2286 cM. The marker density of the present research is higher than that usually adopted in QTL studies based on microsatellite markers: the number of considered markers overcome the presence of only two alleles for each SNP compared to many alleles for each microsatellite marker allowing to define an accurate mapping of the considered regions.

**Table 2:** Average SNPs distance within each of the three analyzed QTLRs

**Chromosome**	**QTLR (cM)**	**QTLR (Mb)**	**SNPs (No)**	**Average distance within each QTLR (Mb)**
1	20 (60–80)	108.9	141	0.77
		(106.9-215.8)		
2	11 (55–66)	45.2	171	0.26
		(32.7-77.9)		
3	18 (42–60)	43.9	67	0.66
		(18.7-62.6)		

### Identification of genes containing SNPs associated with meat pH

The SNPs detected by the *in silico* search were used to identify markers associated with meat pH in the three genomic regions studied. Results highlighted five markers significant at a nominal P-value <0.01 for pH1 (Table 3) and ten markers significant at the same nominal P-value for pHu (Table 4).

**Table 3:** Significant markers detected by association analysis with pH1 values using PLINK

SNP (*)	**Gene symbol**	**Gene name**	**Chromosome**	**Mb**	**P**	
					**UNADJ**	**FDR**
8E_018	*KDM3A*	lysine-specific demethylase 3A	3	61.06	0.000837	0.09081
8E_018a			3	61.06	0.000837	0.09081
2M_060	*EPB42* / *LOC100525673*	E3 ubiquitin-protein ligase UBR1-like	1	143.13	0.006212	0.352
2M_059a			1	143.13	0.00659	0.352
2M_040	*SPINT1*	serine peptidase inhibitor, Kunitz type 1	1	145.62	0.009615	0.352

(*) in this column the laboratory codes of the SNPs identified within UniGene cluster selected are indicated.

UNADJ = nominal P-values.

FDR = P-values after False Discovery Rate correction.

**Table 4:** Significant markers detected by association analysis with pHu values using PLINK

SNP (*)	**Gene**	**Gene name**	**Chromosome**	**Mb**	**P**	
					**UNADJ**	**FDR**
5E_003	*DHPS* / *LOC100519413*	deoxyhypusine synthase-like	2	66.69	8.926e-005	**0.01937**
5M_105	*LOC100513647*	uncharacterized *LOC100513647*	2	65.38	0.001777	0.173
5M_011	*FARSA* / *LOC100524304*	phenylalanyl-tRNA synthetase alpha chain-like	2	66.28	0.002815	0.173
2M_075	*HERC1*	HECT and RLD domain containing E3 ubiquitin protein ligase family member 1	1	119.56	0.004006	0.173
5M_006	*COL5A3*	collagen, type V, alpha 3	2	69.16	0.005301	0.173
3M_020c	*ACTR10* / *LOC100620619*	actin-related protein 10 homolog	1	208.21	0.007129	0.173
3M_020			1	208.21	0.008583	0.173
5E_019	*BRD2*	bromodomain containing 2	2	61.87	0.008863	0.173
5M_024	*TRMT1*	tRNA methyltransferase 1 homolog (S. cerevisiae)	2	66.24	0.00914	0.173
5M_032	*MAN2B1* / *LOC100518647*	lysosomal alpha-mannosidase-like	2	66.72	0.009565	0.173

(*) in this column the laboratory codes of the SNPs identified within UniGene cluster selected are indicated.

UNADJ = nominal P-values.

FDR = P-values after False Discovery Rate correction.

The P-value significant after FDR correction is indicated in bold.

Out of the five markers associated with pH1 values, two SNPs detected on the same gene were mapped on SSC3 and three were identified on SSC1. Two of the SNPs matched two different UniGene clusters but they correspond to the same gene. On the whole, the five SNPs associated to pH1 were detected in three genes that are listed here from the most significant to the less significant: *KDM3A*, *EPB42* / *LOC100525673*, *SPINT1*. On Table 3 the gene names, their chromosome localization and their position on the genomic sequence are reported. The markers associated with pHu values were mapped on SSC1 and SSC2 and the three most significant located on chromosome 2. The genes corresponding to the markers associated to pHu were (from the most significant to the least significant): *DHPS* / *LOC100519413*, Uncharacterized *LOC100513647*, *FARSA* / *LOC100524304*, *HERC1*, *COL5A3*, *ACTR10* / *LOC100620619*, *BRD2*, *TRMT1*, *MAN2B1* / *LOC100518647*. On Table 4 the gene names, their chromosome localization and their position on the genomic sequence are reported.

After FDR correction for multiple testing only the SNP related to pHu that was found in the *DHPS* gene remained significant (P = 0.01937).

Using this marker we genotyped the Group 2 of pigs to analyze the additive effect of the SNP on the studied trait. The most frequent genotype was the homozygous TT (228 out of 311 tested pigs) while the frequency of the rarest C allele was 0.15 (Table 5). The TT pigs showed lower values of pHu than CC and TC animals and the difference with the other homozygous group (CC) was of 0.13 unit of pH (additive effect of 0.065 pHu unit, P < 0.05). In view of the scarce number of CC genotypes detected (N = 11) we performed an additional analysis considering together the TC and CC genotypes. This analysis confirmed the previous results showing a difference between the TT and the TC + CC pigs (data not shown; Additional file 1: Table S1). To detect which part of the total variance of meat pHu was explained by the significant markers we compared the R^2^ values obtained by a GLM including the marker in the model (R^2^ = 0.18) with those calculated without the marker in the model (R^2^ = 0.15). The difference between these two values (0.03) represent the proportion of the variance of pork ultimate pH explained by the SNP.

**Table 5:** **Association analysis of 5E_003 (*DHPS *) SNP to pHu**

**No**	**F**	**P**	**LSM ± SE**			**Additive effect**	**P (Add.)**	**Dominance effect**
			**TT (No)**	**TC (No)**	**CC (No)**			
311	4.71	0.010	5.731 ± 0.024^a^ (228)	5.808 ± 0.033^b^ (72)	5.866 ± 0.068^b^ (11)	0.068 ± 0.034	0.045	NS

a, b: Different superscript letters indicated differences in the estimated pHu values between pairs of genotype class significant at P < 0.05.

F = value of the Fisher test.

P = probability of the Fisher test.

LSM = Leas Square Means.

SE = standard error.

NS = not significant.

### Genomic characterization of the most significant SNP detected

**Figure 1:**
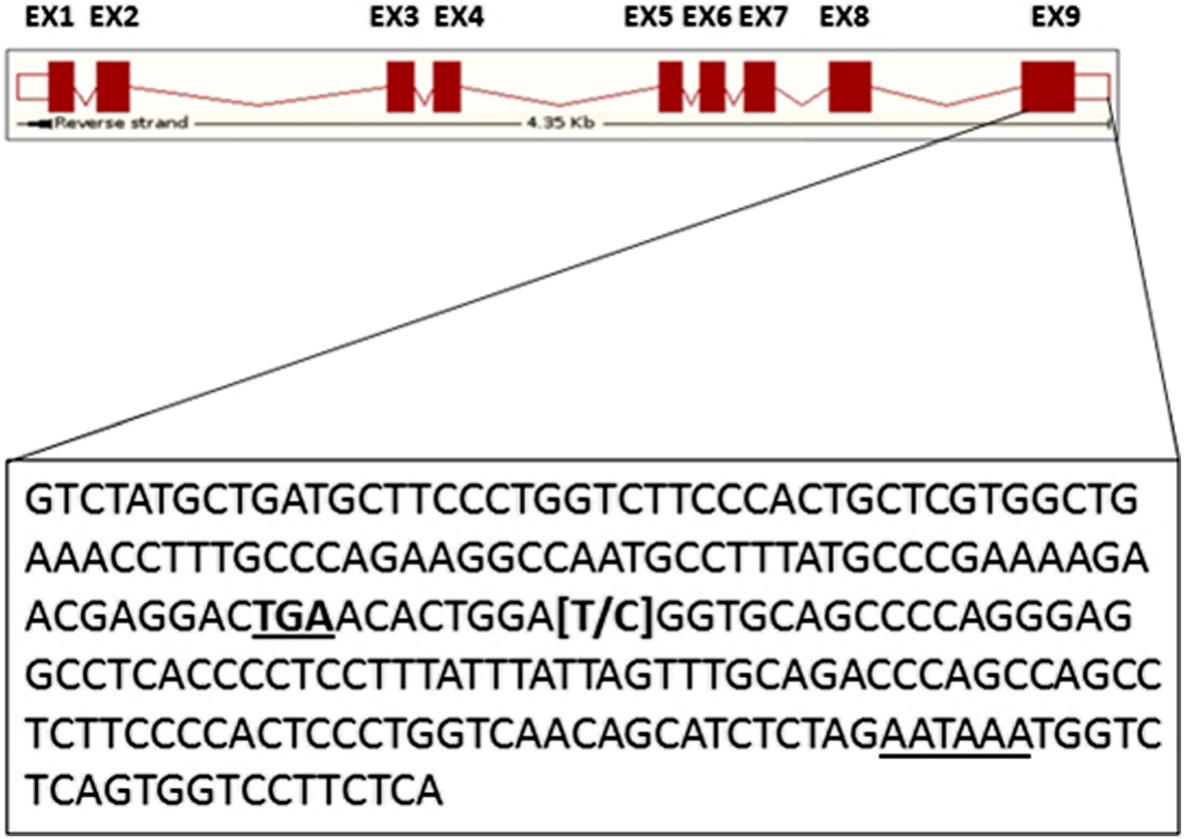
**Exon-intron structure of *DHPS *gene.** The position of the detected SNP (g. 66,686,842 T > C) in the 3’ untranslated region present in exon 9 is indicated in square brackets. The stop codon and the polyadenylation signal are underlined.

By checking the UniGene cluster in which the SNP was detected, we found that the gene was Deoxyhypusine synthase-like (*DHPS*). The analysis of *Sus scrofa* 10.2 genomic sequence allowed to found that the polymorphism was located on the ninth and last exon of the gene within the 3’ untranslated region (3’UTR), nine nucleotides after the stop codon (Figure 1). The point mutation detected was located at nucleotide 66,686,842 of the current sequence of porcine chromosome 2 (g.66686842 T > C). This gene is a catalyzer of the first step of a posttranslational modification characterized by the transfer of the aminobutyl moiety of polyamine spermidine to one specific lysine residue of eIF5A precursor, to form an intermediate deoxyhypusine residue [14]. This intermediate product is hydroxylated in a second reaction by Deoxyhypusine hydroxylase/monooxygenase (*DOHH*) to obtain Hypusine [Nϵ-(4-amino-2-hydroxybutyl1)-lysine] - eIF5A complex [15,16].

**Figure 2:**
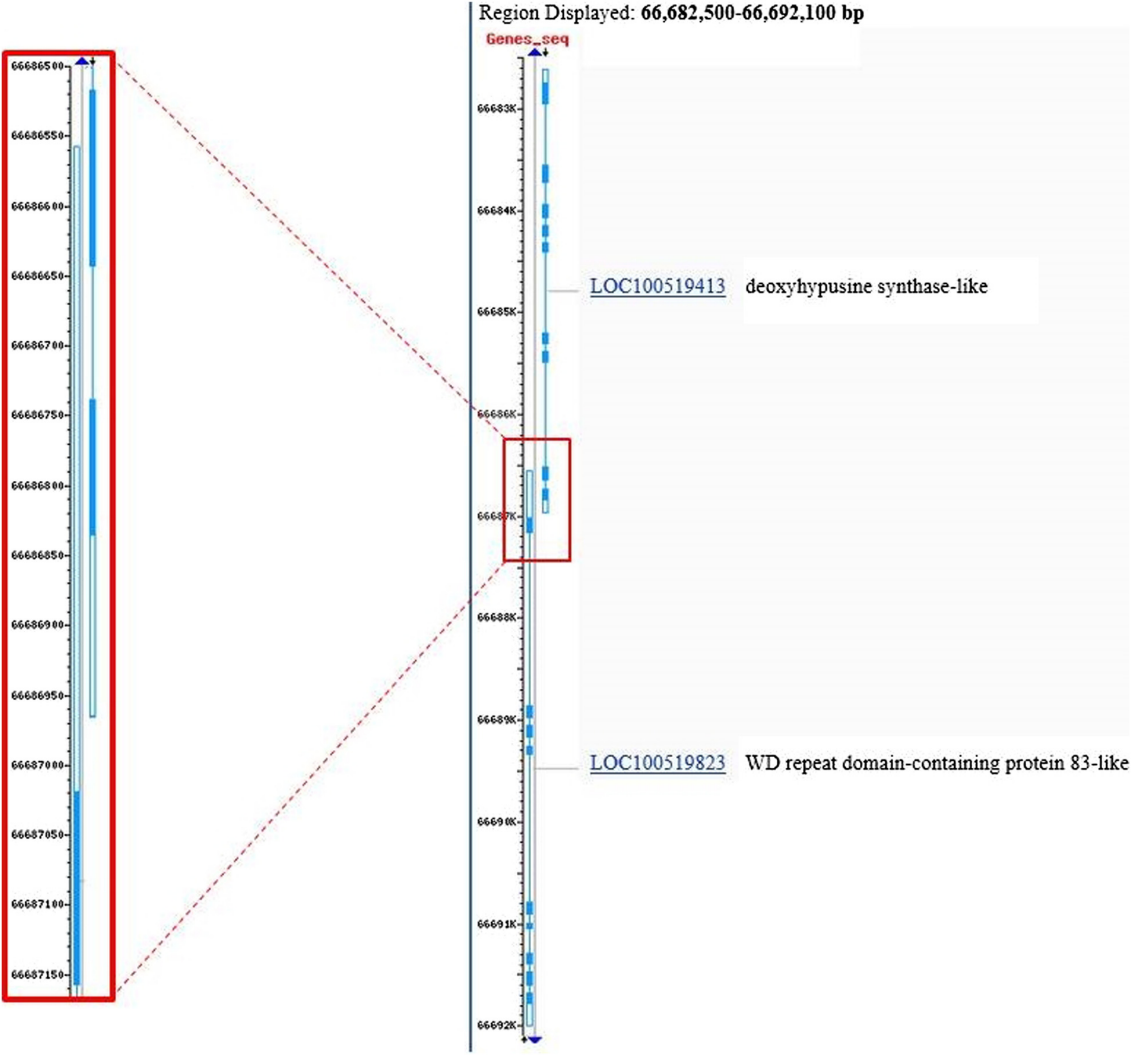
**Complexity of the region of *DHPS *and *WDR83 *genes in pigs.** The two genes *DHPS* and *WDR83* are coded on opposite strands of porcine chromosome 2 between 66.6 and 66.7 Mb of the porcine genomic sequence 10.2. A detail of the overlapping final regions of the two genes including the 3’UTRs (white rectangles) and the final part of the coding sequences (pale blue rectangles) are enlarged in the red box.

Visualizing the genomic position of *DHPS* gene using NCBI MapViewer [17] we observed that its 3’end overlaps part of the 3’UTR of another gene, coded on the opposite chromosome strand, WD repeat domain-containing protein 83-like (*WDR83* – *LOC100519823*, Figure 2). *WDR83*, called also *MORG1* (mitogen-activated protein kinase organizer 1) encodes a member of the WD-40 protein family and belongs to a modular scaffold system responsible of the regulation of extracellular signal-regulated kinase (ERK) pathway that has an important role in the modulation of various cellular processes, including gene expression, cell growth, cellular differentiation and apoptosis. *WDR83* interacts also with Egl nine homolog 3 gene (*EGLN3* also known as prolyl-hydroxylase domain-containing protein 3, *PHD3*): an increase in *WDR83* expression activates or stabilizes the *EGLN3* mRNA level [18]. The latter gene, *EGLN3*, in normoxic conditions, hydroxylates the product of hypoxia-inducible-factor 1 alpha subunit (basic helix-loop-helix transcription factor) gene (*HIF1A*) allowing the degradation of HIF1 alpha protein. On the contrary, at lower oxygen concentration *WDR83* expression decreases and *EGLN3* activity is reduced with the consequence of a higher stability of *HIF1A* mRNA allowing the activation of downstream metabolic processes essentials to reduce the effect of low oxygen level in tissues [19,20] as angiogenesis, erythropoiesis, increased expression of glycolytic enzymes and glucose transporters to produce more energy (ATP) from anaerobic glycolysis.

In humans the genes *DHPS* and *WDR83* are included in the group for which was reported a bidirectional regulation of mRNA stability by the natural antisense transcript (NAT) method of regulation. In particular, *WDR83* and *DHPS* are an example of cis-NAT regulation i.e. the two transcripts are partially overlapping in their 3’UTRs, coded in opposite direction by the same DNA stretch [18]. With this system of regulation the mRNA expression and proteins levels are regulated concordantly. The NAT method of regulation was identified in several mammalian genomes [18,21]. NATs principal functions are related to the regulation of the expression of sense transcripts, the hybridization with them, and to influence mRNA transcription or stability [21,22]. Other roles proposed for NATs are an involvement in DNA methylation, chromatin modification and mono allelic expression. Researches concerning these two genes are often related to cancer biology [18,23,24].

Little is known about the role of these two genes in tissues like skeletal muscle but a possible involvement may be related to the hypoxic conditions occurring in skeletal muscle due to exercise, stress or in post-mortem phase. Hypoxya is a condition that was reported to be present in several conditions: in cancers, when tumor cells grow rapidly their vascular supply become insufficient leading to hypoxia [25,26] but hypoxic condition occurs also in ischemic cardiac myocytes [27] and in skeletal muscle under exercise [28] and in post mortem. The oxygen reduction and the energy deficit of post mortem phase will lead to acidosis due to the anaerobic glycolysis increase that will cause a pH decrease. The complex *DHPS*/*WDR83* is one of the factors modulating *EGLN3* and then *HIF1A*[18] and the polymorphism detected on the common part of the 3’UTR of the two genes may activate this cascade with different efficiency between alleles to cause pH decline in the skeletal muscle cells during post-mortem. In order to validate this hypothesis further researches, aimed to clarify and verify the link of the mutation found in the 3’UTR of *DHPS*/*WDR83* genes with meat pH, are needed before to consider them as functional candidate genes and not only positional markers for the studied trait.

## Conclusions

In the present work we studied QTLRs of 10–20 cM and detected some hundreds of SNPs that allowed a more refined analysis of these regions. Applying FDR to correct for multiple test a SNP found in the 3’UTR of *DHPS*/*WDR83* overlapping genes was found to be associated with the ultimate pH of pig meat. This was the unique SNP showing a significant effect on the studied trait out of the 251 markers used. Nevertheless this result was useful to confirm the localization of the QTL for meat pH reported in literature and allowed to identify genes putatively regulating pork ultimate pH mapping on the QTL region of SSC2. The identified association of the detected marker with meat pH could represent, after confirmation, a new biomarker useful to improve pig meat quality.

## Methods

### Animals, phenotypes and DNA extraction

For this study we sampled a pure-bred population of Italian Large White sib-tested pigs provided by the National Association of Pig Breeders (Associazione Nazionale Allevatori Suini, ANAS) [29]. The pigs farmed at the ANAS genetic station are all tested for the *RYR1* gene (Halothane locus) in order to have the boars selected for the genetic improvement program free from the recessive allele at this locus. For *PRKAG3* gene concerns no genetic test were carried out because in the Italian Large White pig population the negative (dominant) 200Q allele (RN locus) at this locus is not segregating [30]. The animals were reared on the central ANAS Sib-Test station from about 30 kg live weight to about 155 kg live weight. The nutritive level utilised was *quasi ad libitum*, i.e. about 60% of pigs were able to ingest the entire supplied ration. For the genetic evaluation of a boar, full sib triplets (two females and one castrated male) are performance tested. All pigs were slaughtered after electrical stunning in the same commercial abattoir during the year 2008 in 11 different days. Using the 356 SNPs detected in the three QTLRs an association analysis (see PLINK analysis below) on 251 pigs, 170 females and 81 castrated males (Group 1) was performed. The most significant markers were then tested using the 251 animals of Group 1 plus additional 96 samples obtaining a larger group of 347 samples (231 females and 116 castrated males). We refer to this enlarged sample as Group 2. The sex distribution of the animals with a ratio females: castrated males approximately equal to 2 reflects the sex proportion characteristic of the Italian selection scheme described above.

Samples of *semimembranosus* muscle were collected at slaughterhouse from the right ham of the 347 animals and immediately frozen in liquid nitrogen. Genomic DNA was extracted from these samples using a standard protocol [31]. Within about 1 hour *post mortem* the meat pH value (pH1) was recorded on the same muscle. Furthermore, at 24 hours *post mortem* the ultimate pH (pHu) was measured. The statistics of the recorded pH values are reported in Tables 6 and 7.

**Table 6:** **Statistic describing muscle pH1 values measured in *semimembranosus *** **muscle recorded in Italian Large White pigs**

	**Group 1**					**Group 2**				
	No (*)	**Average**	SD (**)	**Min**	**Max**	No (*)	**Average**	SD (**)	**Min**	**Max**
All	248	6.19	0.27	5.37	6.78	347	6.21	0.27	5.37	6.91
Females	167	6.19	0.29	5.37	6.78	231	6.21	0.28	5.37	6.91
Castrated males	81	6.20	0.22	5.72	6.69	116	6.21	0.23	5.72	6.81

(*) pH1 values are not available for all samples.

(**) SD = standard deviation.

**Table 7:** **Statistic describing muscle pHu values measured in *semimembranosus *** **muscle recorded in Italian Large White pigs**

	**Group 1**					**Group 2**				
	**No**	**Average**	SD (**)	**Min**	**Max**	No (*)	**Average**	SD (**)	**Min**	**Max**
All	251	5.76	0.23	5.40	6.65	313	5.77	0.23	5.24	6.65
Females	170	5.73	0.21	5.40	6.47	209	5.73	0.21	5.24	6.47
Castrated males	81	5.84	0.24	5.50	6.65	104	5.84	0.25	5.43	6.65

(*) pHu values are not available for all samples.

(**) SD = standard deviation.

### QTL selection, SNP detection and genotyping

We selected the QTL influencing porcine meat pH by searching in literature and by browsing the PigQTLdb. The most relevant QTL and the correspondent genomic positions were chosen according to these rules: a) significant effect described by different papers and in different swine populations: if a QTL was detected on the same genomic region using different crossing scheme and different breeds it should be relevant for the whole swine species and not limited to a single or few breeds, b) a relevant part of the phenotypic variance explained by the QTL. Only three chromosome regions satisfied both criteria and were selected to be analysed. The extension of the three QTLR we selected was between 11 and 20 cM: SSC1 from 60 to 80 cM, SSC2 from 55 to 66 cM, SSC3 from 42 to 60 cM. A complete list of the literature utilised for the QTL selection is reported on Table 8.

**Table 8:** Genetic crosses and bibliographic references utilized to identify the QTL regions used for this research

**Chromosome**	**Breeds**	**Reference**
SSC1	Duroc x Berlin Miniature	[32]
SSC1	Duroc x Pietrain	[33]
SSC2	Meishan x Pietrain	[34]
SSC2	Large White	[35]
SSC2	Duroc x Landrace	[36]
SSC2	Duroc x Pietrain	[37]
SSC2	Duroc x Pietrain	[33]
SSC2	White Duroc x Erhualian resource population	[38]
SSC2	Commercial crossbred population	[39]
SSC3	Wild Boar x Pietrain	[40]
SSC3	Iberian x Landrace	[41]
SSC3	Duroc x Berlin Miniature	[42]
SSC3	Duroc x Pietrain	[43]

Map intervals were defined by searching for the position of the most significant markers reported in each original paper in the USDA-MARC linkage map [44] that includes all available microsatellite and DNA markers so far analysed. Furthermore, this map is implemented both in PigQTLdb and in NCBI map viewer. In this way it was possible to compare the data contained in both websites. The alignment of porcine and human chromosomes was first performed using pig and human radiation hybrid maps using the tool available within PigQTLdb website, then the aligned regions were visualized using the NCBI map viewer. The obtained output was used to choose in each QTLR all expressed sequences (both mRNAs and expressed sequence tags, ESTs) located in the identified corresponding genomic regions that were grouped according to UniGene clusters. The obtained clusters were filtered to retain only those represented by at least eight sequences, then putative SNPs were searched *in silico* by a multiple alignment of all members of each cluster using BLASTN [45] with the algorithm MegaBLAST. We marked as putative SNP a mutation detected in at least three sequences to avoid inconsistencies due to sequencing errors and also to exclude SNPs with a rare allele. Moreover, the obtained multiple alignments were manually scored in order to detect those suitable to be analysed by the high throughput Illumina GoldenGate Genotyping Assay system [46]. When more than one polymorphism was detected within each cluster we discarded those closer more than 80 nucleotides because they were not suitable to design the probes to be used with this genotyping system. These SNPs were finally scored with the specific Illumina software to establish the SNP score of each sequence used to calculate the parameter indicated as designability rank. In this way we obtained a customized array of new SNPs detected in the transcribed region of messenger RNAs. Genotyping of 251 samples of Group 1 was carried out by an outsource company (CBM, Cluster in biomedicine, Trieste, Italy, [47]). Genotypes of the samples included in Group 2 were obtained by High Resolution Melting (HRM), that is an efficient technique to determine a genotype using the melting profile of small amplicons [48,49]. For this aim, primers flanking the polymorphism were designed, to amplify a 181 bp fragment (For: 5’- GCCCGAAAAGAACGAGGA -3’, Rev: 5’- ACCCACTACCAAGGACACAGA -3’). Amplifications were performed with Rotor-Gene TM 6000 (Corbett Research, Mortlake, New South Wales, Australia), in a total volume of 20 μl containing 2 μl of 10× standard buffer, 3 mM MgCl2, 0.3 μM of each primer, 160 μM dNTP, 1 U EuroTaq polymerase, 1 U EvaGreen TM (Biotium Inc., Hayward, CA, USA) and 50–100 ng of template DNA. Cycling conditions were: initial denaturation at 95°C for 5 min, 40 cycles of 95°C for 30 s, 56°C for 15 s and 72°C for 2 min, followed by a final extension step of 72°C for 2 min. Fluorescence was acquired at the end of each extension step to ensure that all reactions reached the plateau stage. After a holding step at 50°C for 60 s, a HRM analysis was performed heating the samples from 83 to 88°C, at a rate of 0.1°C each 4 s, with continuous fluorescence acquisition. The HRM data were analysed by Rotor-Gene TM 6000 software. Fluorescence vs. temperature plots were normalized by selecting linear regions before and after the melting transition. Genotypes were determined setting known genotypes samples as reference and using a reliability threshold of 0.90 for the genotype assignment.

### Statistical analyses

The association study including the markers detected within the analysed QTLRs was performed with PLINK whole genome association analysis toolset [50,51]. The genotypes of animals belonging to Group 1 were filtered before the association analysis. All markers having a minor allele frequency below 0.01 (N = 162) were discarded. Furthermore Hardy-Weinberg equilibrium was tested and four SNPs were discarded because not in equilibrium (p < =0.01). After filtering, 218 markers and all 251 individuals (Group 1) were retained. To correct for stratification of the considered population a clustering method based on an identical by state (IBS) approach included in PLINK was used. Furthermore, a stratified association analysis was performed using the Cochran-Mantel-Haenszel test implemented in PLINK. The significant markers were further assayed for multiple testing using the False Discovery Rate (FDR) correction using as significance threshold P < 0.05.

The significant marker detected by PLINK was further analysed to validate the association between pH values and the genotypes scored on Group 2 of pigs using the MIXED procedure of SAS release 9.2 (SAS, Institute Inc., Cary, NC). The model included genotype of the analysed markers, sex, and day of slaughter as fixed effects and sire as random effect:

$$\mathrm{pHu}=µ+{\mathrm{SNP}}_{i}+{\mathrm{Sex}}_{j}+{\mathrm{Day}}_{k}+\mathrm{Sire}+\epsilon_{\mathrm{ij}}{}_{k}$$

where: pHu = ultimate pH; μ = overall mean; SNP = fixed effect of each genotype (i = 1–3); Sex = fixed effect of sex (j = 1,2); Day = fixed effect of day of slaughter (k = 1–11); Sire = random effect of the sire; ϵ = residual error.

Finally, the GLM procedure of SAS release 9.2 (SAS, Institute Inc., Cary, NC) was used to calculate the part of total variance explained by the SNP (R^2^). In order to estimate the proportion of the genetic variance explained by the analysed SNP we compared the R^2^ of a fixed model including genotypes of the marker, sex, and day of slaughter with a reduced model without genotypes including as fixed effects sex, and day of slaughter. The difference between the two R^2^ indicates the total variance of pork ultimate pH explained only by the SNP.

The first model was:

$$\mathrm{pHu}=µ+{\mathrm{SNP}}_{i}+{\mathrm{Sex}}_{j}+{\mathrm{Day}}_{k}+\epsilon_{\mathrm{ij}}{}_{k}$$

where: pHu = ultimate pH; μ = overall mean; SNP = fixed effect of each genotype (i = 1–3); Sex = fixed effect of sex (j = 1,2); Day = fixed effect of day of slaughter (k = 1–11); ϵ = residual error.

The second model was:

$$\mathrm{pHu}=µ+{\mathrm{Sex}}_{i}+{\mathrm{Day}}_{j}+\epsilon_{\mathrm{ij}}$$

where: pHu = ultimate pH; μ = overall mean; Sex = fixed effect of sex (i = 1,2); Day = fixed effect of day of slaughter (j = 1–11); ϵ = residual error.

## Competing interests

The authors declare that they have no competing interests.

## Authors’ contributions

PZ performed the *in silico* and statistical analyses and drafted the manuscript; MB, LFdP and SB carried out the molecular genetic analysis; LB and MG contributed providing samples and genetic indexes; LB contributed to the statistical analyses of the data; VR and RD participated in the design of the study, coordinated the project and contributed both to draft and to revise the manuscript. All authors read and approved the final manuscript.

## Supplementary Material

Additional file 1: Table S1 Association analysis of 5E_003 (DHPS) to pHu SNP performed using two genotype classes. Click here for file
