# Supplementary material for: SNPs detection in DHPS-WDR83 overlapping genes mapping on porcine chromosome 2 in a QTL region for meat pH
Source: BMC Genet. 2013 Oct 8;14:99. doi: 10.1186/1471-2156-14-99 (PMC4124853; doi:10.1186/1471-2156-14-99)
Supplement: Additional file 1: Table S1 — Association analysis of 5E_003 (DHPS) to pHu SNP performed using two genotype classes. [file 1471-2156-14-99-S1.docx]

**Supplementary Table 1. Association analysis of 5E_003 (DHPS) to pHu SNP performed using two genotype classes.**

The genotypes TT were compared with the genotypes TC+CC, considered as a single class.

| No | P | LSM ± SE | |
| --- | --- | --- | --- |
|  |  | TT (No) | TC+CC (No) |
| 311 | 0.0035 | 5.731 ± 0.024  (228) | 5.816±0.032  (83) |

P = probability of the Fisher test

LSM = Leas Square Means

SE = standard error
